# Connexin43 recruits PTEN and Csk to inhibit c-Src activity in glioma cells and astrocytes

**DOI:** 10.18632/oncotarget.10454

**Published:** 2016-07-06

**Authors:** Ana González-Sánchez, Myriam Jaraíz-Rodríguez, Marta Domínguez-Prieto, Sandra Herrero-González, José M. Medina, Arantxa Tabernero

**Affiliations:** ^1^ Instituto de Neurociencias de Castilla y León (INCYL), Departamento de Bioquímica y Biología Molecular, Universidad de Salamanca, Salamanca, Spain

**Keywords:** connexin, Src, glia, CNS, gap junctions

## Abstract

Connexin43 (Cx43), the major protein forming gap junctions in astrocytes, is reduced in high-grade gliomas, where its ectopic expression exerts important effects, including the inhibition of the proto-oncogene tyrosine-protein kinase Src (c-Src). In this work we aimed to investigate the mechanism responsible for this effect. The inhibition of c-Src requires phosphorylation at tyrosine 527 mediated by C-terminal Src kinase (Csk) and dephosphorylation at tyrosine 416 mediated by phosphatases, such as phosphatase and tensin homolog (PTEN). Our results showed that the antiproliferative effect of Cx43 is reduced when Csk and PTEN are silenced in glioma cells, suggesting the involvement of both enzymes. Confocal microscopy and immunoprecipitation assays confirmed that Cx43, in addition to c-Src, binds to PTEN and Csk in glioma cells transfected with Cx43 and in astrocytes. Pull-down assays showed that region 266–283 in Cx43 is sufficient to recruit c-Src, PTEN and Csk and to inhibit the oncogenic activity of c-Src. As a result of c-Src inhibition, PTEN was increased with subsequent inactivation of Akt and reduction of proliferation of human glioblastoma stem cells. We conclude that the recruitment of Csk and PTEN to the region between residues 266 and 283 within the C-terminus of Cx43 leads to c-Src inhibition.

## INTRODUCTION

Connexin43 (Cx43) is an integral membrane protein that assembles to form gap junction channels and hemichannels in different cell types, including astrocytes, where Cx43 is strongly expressed [1, 2]. However, the levels of Cx43 protein are decreased when these cells acquire a malignant phenotype. In fact, the levels of Cx43 protein are inversely correlated with the degree of malignancy in astrocytomas, being negligible in the majority of glioblastomas, the most common glioma that unfortunately carries the worst prognosis [3–9]. Even in glioma-initiating cells or glioblastoma stem cells (GSCs)-a subpopulation of cells within malignant gliomas that are characterized by their self-renewal capacity, multilineage differentiation properties, high oncogenic potential, and resistance to standard therapies [10]- the levels of Cx43 are negligible [11, 12].

The proto-oncogene tyrosine-protein kinase Src (c-Src) participates in signaling pathways that control a diverse spectrum of biological events, including proliferation, differentiation, survival and migration [13]. c-Src binds to Cx43 through the Src homology 3 (SH3) domain binding motif of Cx43, a proline-rich region (amino acids 274–283), and then phosphorylates tyrosine 265, providing an SH2 domain binding site with subsequent phosphorylation at tyrosine 247 [14]. As a consequence of these phosphorylations, gap junctional intercellular communication is reduced [15–17], and Cx43 turnover is initiated [18]. More recently, it has been found that in addition to these effects, the interaction of Cx43 with c-Src can reciprocally inhibit c-Src activity [11, 19]. Glioblastoma cells exhibit strong c-Src activity [20], which plays an important role in the transforming phenotype of astrocytomas [21]. Autophosphorylation at tyrosine 416 activates c-Src, contributing to the malignant phenotype [22]. Interestingly, restoring Cx43 to glioma cells reduces c-Src activity by decreasing the active form of c-Src (c-Src phosphorylated at tyrosine 416; Y416 c-Src) and increasing the inactive form of c-Src (c-Src phosphorylated at tyrosine 527; Y527 c-Src) [19]. The inhibition of c-Src is not promoted by mutant Cx43, which lacks the ability to bind to c-Src [19]. Rather, it is promoted by cell-penetrating peptides containing the region of Cx43 involved in c-Src interaction [11]. Collectively, these data indicate that the interaction of Cx43 with c-Src reduces c-Src activity; therefore, these proteins are mutually regulated by a phosphorylation/dephosphorylation loop. c-Src activity is linked to crucial signaling pathways [23]. Consequently, it is not surprising that the inhibition of c-Src promoted by Cx43 reduces the cell cycle [11, 19], glucose uptake [24, 25] or glioma stem cell phenotype [11, 19] (for a review, see [26]).

Despite the relevance of c-Src inhibition for cell biology, the mechanism by which the interaction of Cx43 with c-Src reduces its activity is unknown. The inhibition of c-Src requires the activity of the C-terminal c-Src kinase (Csk), which phosphorylates c-Src at tyrosine 527 [27]. In addition, several phosphatases, such as phosphatase and tensin homolog (PTEN), have been shown to dephosphorylate c-Src at tyrosine 416 [28], an activity that is required to complete the inactivation of c-Src. In this study, we found that a small region located in the C-terminal domain of Cx43 serves as a docking platform for c-Src, PTEN and Csk, favoring the inhibition of the oncogenic activity of c-Src.

## RESULTS

### Csk and PTEN are involved in the inhibition of glioma cell growth promoted by Cx43

**Figure 1:**
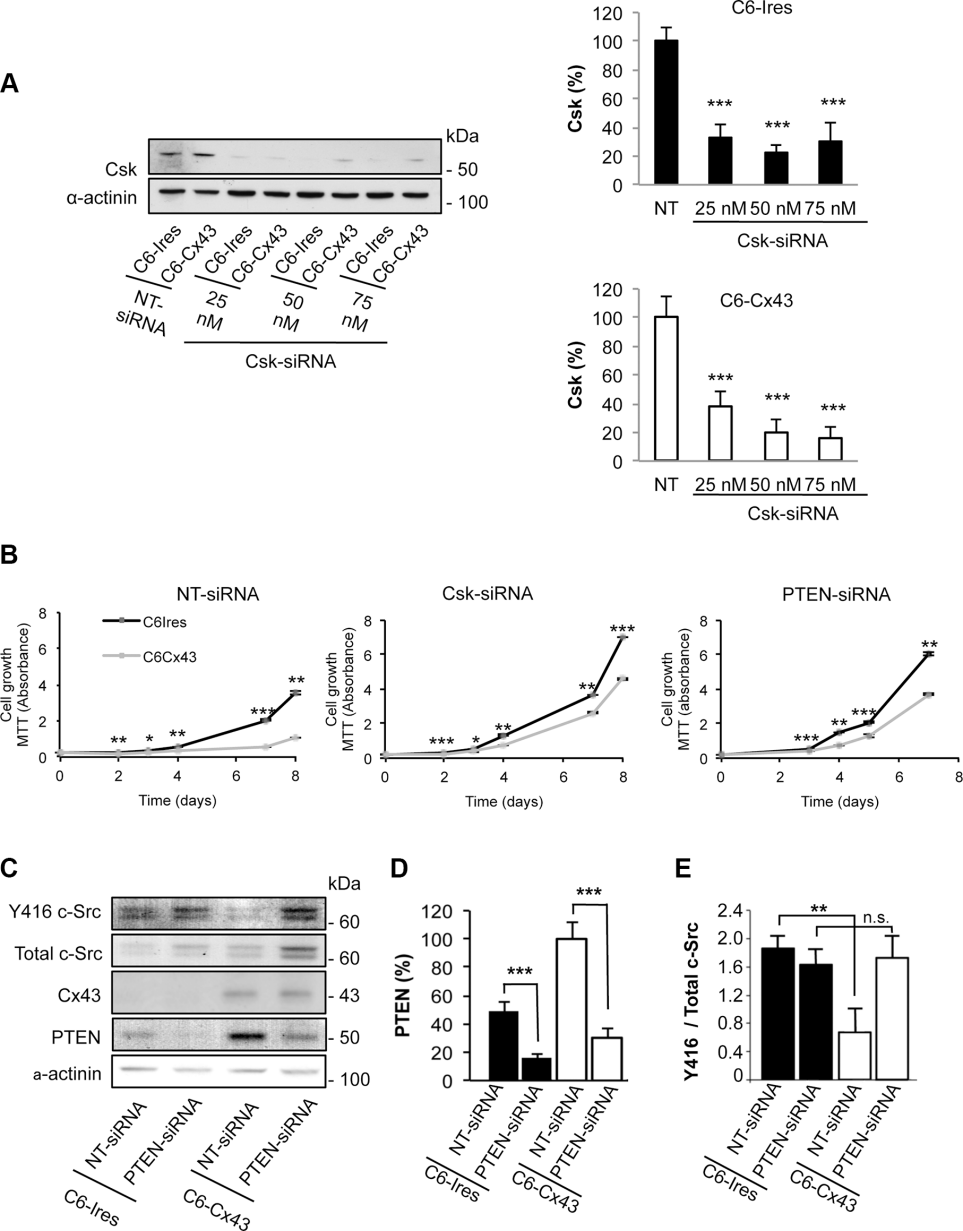
Effect of silencing Csk and PTEN on the reduction of proliferation promoted by Cx43 C6 cells stably transfected with the empty vector (C6-Ires) or the vector containing the Cx43 cDNA (C6-Cx43) were transfected with Csk-siRNA, PTEN-siRNA or non-targeting siRNA (NT-siRNA). (**A**) The levels of Csk were analyzed by Western blotting 72 h after transfection with increasing concentrations of Csk-siRNA or 50 nM NT-siRNA. The results are the means ± s.e.m. (*n* = 3) and they are expressed as the percentage of the corresponding NT-siRNA. ***p < 0.001 versus the corresponding *NT-siRNA*. (**B**) The cells transfected with the indicated siRNAs were plated at 2000 cells/cm^2^, and the number of living cells was followed by the MTT assay. The results are the means ± s.e.m. (*n* = 4) and they are expressed as the absorbance values of the MTT assay. *p < 0.05, **p < 0.01, ***p < 0.001; C6-Ires versus C6-Cx43. (**C**) Cells were transfected with 50 nM NT-siRNA or PTEN-siRNA. After 48 h, PTEN, Cx43, total c-Src and Y416 c-Src levels were analyzed by Western blotting. The results are the means ± s.e.m. (*n* = 6) and they are expressed as the percentage of the C6-Cx43 NT-siRNA (**D**) or as the ratio of Y416 c-Src/total c-Src (**E**). n.s: not significant. **p < 0.01 and ***p < 0.001.

In previous studies, we showed that restoring Cx43 to glioma cells inhibits c-Src activity and consequently their rate of proliferation [11, 19]. Because Csk, through phosphorylation of c-Src at tyrosine 527, is the main enzyme responsible for c-Src inhibition [27], in this study, we addressed the participation of Csk in the antiproliferative effect of Cx43. To do so, the expression of Csk was knocked-down by a specific siRNA (Csk-siRNA) [29] in C6 glioma cells stably transfected with Cx43 (C6-Cx43) or the empty vector (C6-Ires). Figure 1A shows that Csk-siRNA strongly reduced the expression of Csk in both C6-Ires and C6-Cx43 cells at concentrations ranging from 25 to 75 nM. Next, the growth of C6-Ires and C6-Cx43 cells, transfected with 50 nM non-targeting siRNA (NT-siRNA), or Csk-siRNA, was followed using the MTT assay. Figure 1B shows that, as expected, restoring Cx43 reduced the rate of glioma cell growth in the control situation (cells transfected with NT-siRNA). However, when Csk was silenced the effect of Cx43 on glioma cell proliferation was reduced, suggesting the contribution of Csk to the antiproliferative effect of Cx43. It should be mentioned that the growth rate increased when Csk was silenced in both C6-Ires (*p* < 0.01 at days 3 and 4, and *p* < 0.001 for the other days) and C6-Cx43 (*p* < 0.05 at day 3 and *p* < 0.001, for the other days) cells compared with cells transfected with NT-siRNA.

In addition to the phosphorylation at tyrosine 527 by Csk, to be completely inactivated, c-Src needs to be dephosphorylated at tyrosine 416. Several phosphatases, including PTEN, have been shown to dephosphorylate c-Src at tyrosine 416 [28]. To investigate the participation of PTEN in the inhibition of c-Src promoted by Cx43 [19], the levels of Y416 c-Src were analyzed in glioma cells in which PTEN was knocked-down by siRNA [30] (Figure 1C–1E). As expected, Cx43 decreased the ratio of Y416 c-Src / total c-Src in glioma cells transfected with NT-siRNA (Figure 1E). By contrast, when PTEN was silenced by a specific siRNA (PTEN-siRNA), Cx43 could not reduce this ratio (Figure 1E), suggesting that PTEN is involved in the dephosphorylation of c-Src at tyrosine 416. Next, we investigated the participation of PTEN in the antiproliferative effect of Cx43. Our results showed that silencing PTEN reduced the antiproliferative effect of Cx43 on glioma cells compared with glioma cells transfected with NT-siRNA (Figure 1B). The growth rate increased when PTEN was silenced in both C6-Ires (*p* < 0.01 at days 3 and 7, and *p* < 0.001 for the other days) and C6-Cx43 (*p* < 0.01 at days 3 and 7, and *p* < 0.001 for the other days) cells compared with cells transfected with NT-siRNA.

### Restoring Cx43 expression in glioma cells increases PTEN by a c-Src-dependent mechanism

**Figure 2:**
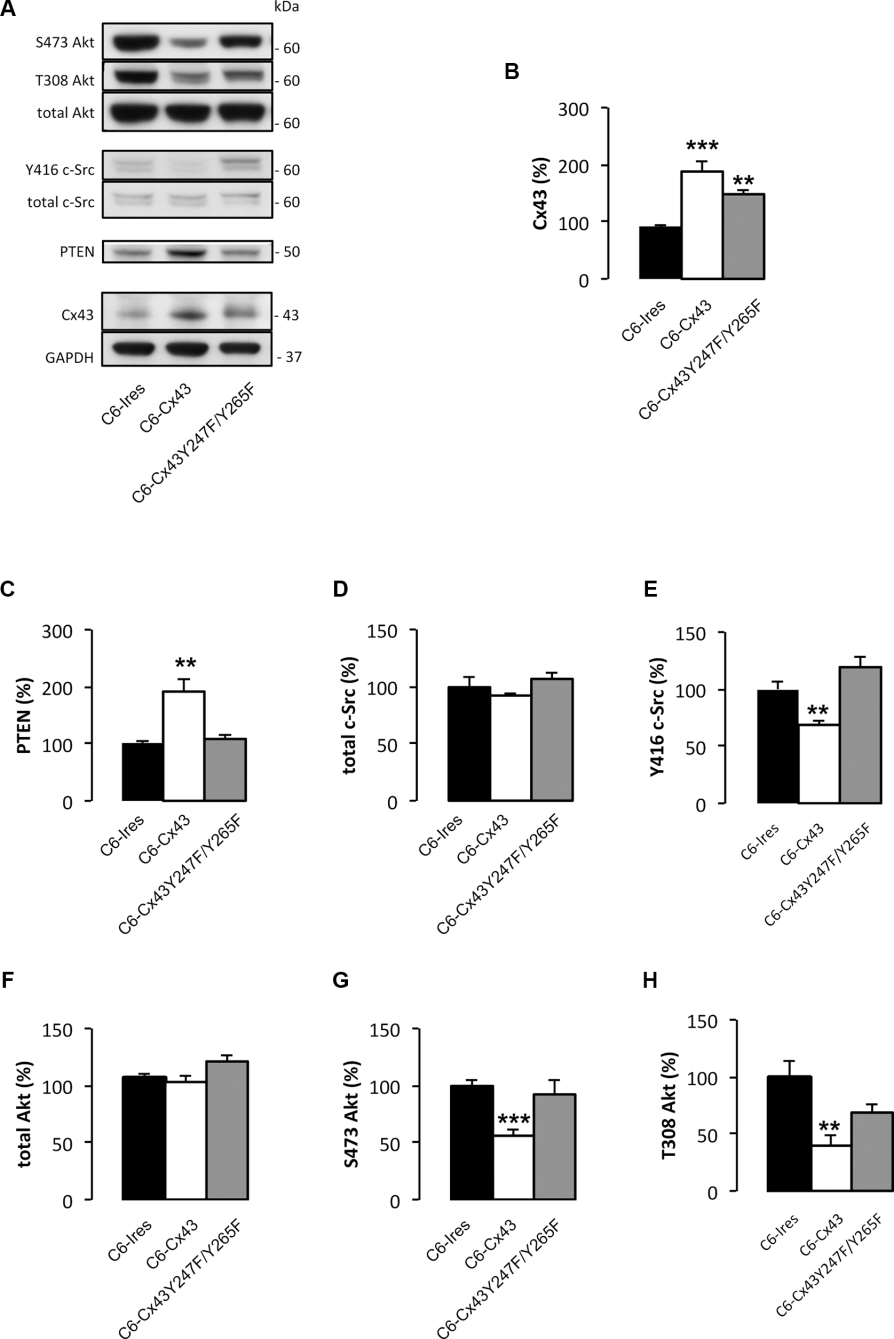
Effect of Cx43 and mutant Cx43 expression on PTEN expression and Akt activity C6 glioma cells were stably transfected with the empty vector (C6-Ires), the vector containing the Cx43 cDNA (C6-Cx43), or the vector containing the cDNA encoding the double mutant Cx43 in which tyrosine 247 and tyrosine 265 of Cx43 were replaced by non-phosphorylatable phenylalanines (C6-Cx43 Y247F/Y265F). Western blots (**A**) and quantification of Cx43 (**B**), PTEN (**C**), total c-Src (**D**), Y416 c-Src (**E**), total Akt (**F**), S473 Akt (**G**) and T308 Akt (**H**). The results are the means ± s.e.m. (*n* = 6) and they are expressed as percentage of the value generated by C6-Ires cells. *p < 0.05, **p < 0.01, ***p < 0.001 versus C6*-*Ires.

Unexpectedly, we observed that the presence of Cx43 increased PTEN protein levels in glioma cells (Figure 1C). Because PTEN is one of the most relevant tumor suppressor proteins in gliomas [31], we decided to explore this effect. To this end, the levels of PTEN were analyzed in C6-Ires and C6-Cx43 cells. Our results showed that restoring Cx43 expression doubled the levels of PTEN protein in glioma cells (Figure 2A and 2C).

To confirm the functionality of PTEN, we analyzed its downstream pathway. PTEN exhibits both lipid and protein phosphatase activities. The main substrate for the lipid phosphatase activity is phosphatidylinositol-trisphosphate (PIP3), which is dephosphorylated by PTEN to generate PIP2. PIP3 is the main activator of Akt [32]. Thus, the higher the activity of PTEN, the lower the activity of Akt. In agreement with this concept, restoring Cx43 expression reduced Akt activity as shown by the concomitant reduction in the levels of Akt phosphorylated at serine 473 and threonine 308 (Figure 2A, 2G and 2H) and the increase in PTEN levels (Figure 2A and 2C).

Because the activity of c-Src promotes PTEN degradation and reduces its ability to inhibit the PI3K/Akt pathway [33], we analyzed the involvement of c-Src in the effect of Cx43 on PTEN. C6 cells were stably transfected with a double Cx43 mutant in which tyrosines 247 and 265 were replaced with non-phosphorylatable phenylalanines (C6-Cx43Y247F/Y265F). This mutant lacks the ability to bind to c-Src [15, 17] and, consequently, does not inhibit c-Src activity like wild-type Cx43 [19] (Figure 2A, 2D and 2E). Our results showed that the mutant Cx43 did not significantly modify the levels of PTEN (Figure 2A and 2C), suggesting that the Cx43-mediated inactivation of c-Src is required to increase the levels of PTEN. In agreement with the lack of effect on PTEN levels, mutant Cx43 did not significantly modify the levels of active Akt phosphorylated at serine 473 and threonine 308 (Figure 2A, 2G and 2H).

### Cx43 binds to PTEN and Csk

**Figure 3:**
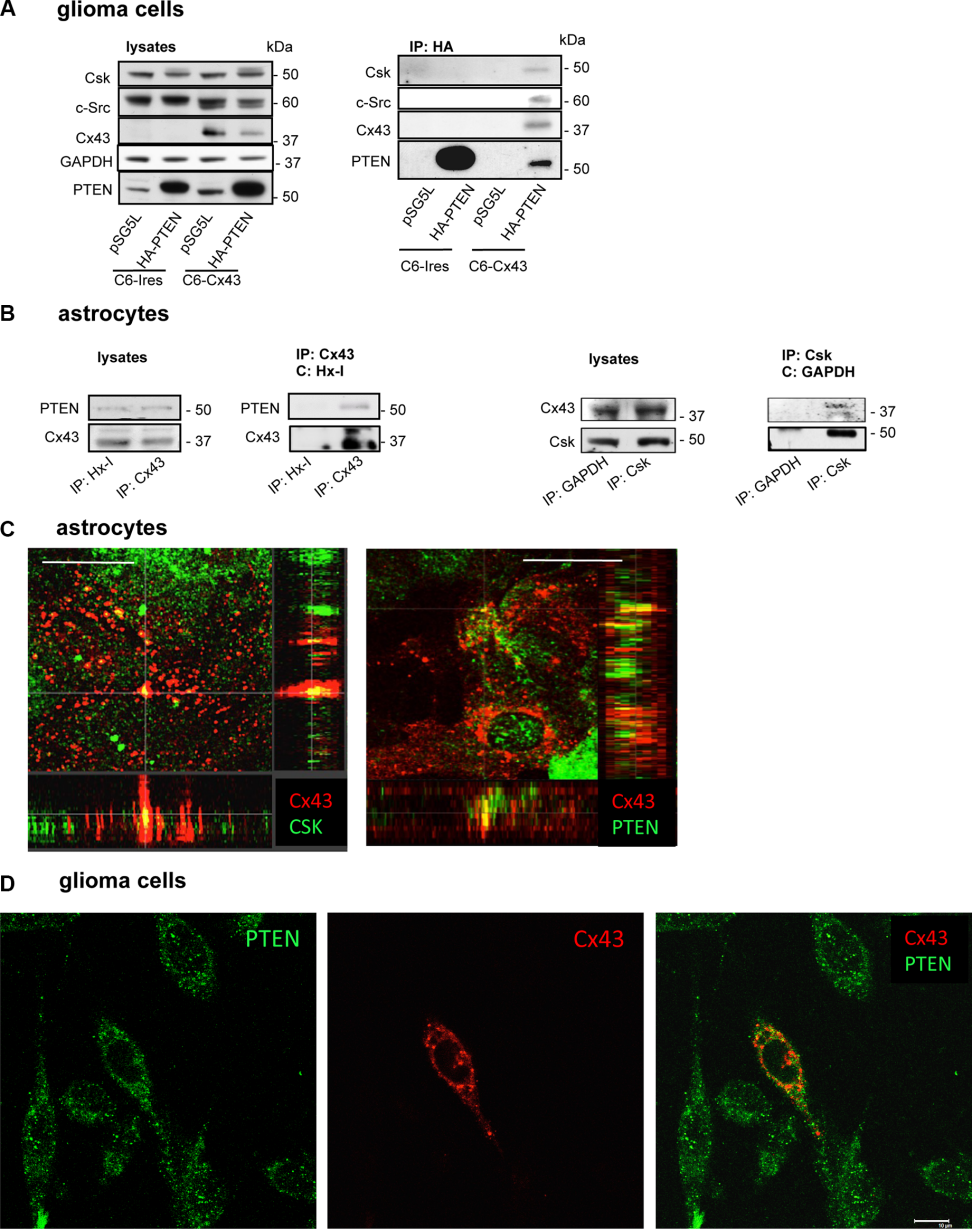
Interaction between Cx43, c-Src, Csk and PTEN in C6 glioma cells and astrocytes (**A**) C6 glioma cells stably transfected with the empty vector (C6-Ires) or the vector containing the Cx43 cDNA (C6-Cx43) were transfected with HA-PTEN or pSG5L (empty vector). After 24 h, the cells were lysed and immunoprecipitated with anti-HA antibodies. Western blotting before (lysates) and after HA immunoprecipitation for PTEN, Cx43, c-Src and Csk showing the presence of Csk, c-Src and Cx43 in the immunocomplex obtained with HA-PTEN in glioma cells expressing Cx43. (**B**) Astrocytes from primary culture were lysed and immunoprecipitated with antibodies against Cx43 or Csk. Antibodies against hexokinase-1 (Hx-1) and GAPDH were used as controls. (**C**) Colocalization of Cx43 with Csk and PTEN in astrocytes. Confocal images show Cx43 (red) and Csk or PTEN (green) and their colocalization (yellow). Scale bars: 15 μm. Orthogonal projections along the z-axis of the images are shown at the bottom and right. (**D**) Colocalization of PTEN and Cx43 in C6 glioma cells transiently transfected with the construct containing Cx43 cDNA. The confocal images show PTEN (green), Cx43 (red) and overlay image with some points of colocalization (yellow). Note that only one Cx43-transfected cell is shown. Scale bar: 10 μm.

The ability of Cx43 to bind to c-Src has been well described [15, 17, 34]. Because our results suggested that Csk and PTEN participate in the inhibition of c-Src activity promoted by Cx43, we postulated that Cx43 could recruit these enzymes to achieve the inactivation of c-Src. To test this hypothesis, glioma cells were transfected with HA-PTEN or the empty vector (pSG5L). After 24 h, HA was immunoprecipitated (Figure 3A). Our results showed that the antibody against HA precipitated HA-PTEN, Cx43, c-Src and Csk in glioma cells transfected with Cx43 but not in glioma cells that lacked Cx43 expression (C6-Ires) or in C6-Cx43 cells transfected with pSG5L, suggesting that restoring Cx43 to glioma cells promotes its binding to PTEN, c-Src and Csk in glioma cells.

To address whether this interaction occurs between endogenously expressed proteins, astrocytes, which naturally express high levels of Cx43 and PTEN, were analyzed. Thus, Cx43 was immunoprecipitated, and the presence of PTEN was identified in the immunocomplex of Cx43 (Figure 3B). Reciprocally, Cx43 was also found after immunoprecipitation with antibodies against Csk in astrocytes. No signals were observed after the immunoprecipitation without IgG (data not shown) or with non-relevant IgGs, such as a mouse monoclonal antibody against hexokinase-1 (Hx-1) or glyceraldehyde-3-phosphate dehydrogenase (GAPDH) used as negative controls.

To confirm the results of immunoprecipitation, immunofluorescence analyses were carried out for Cx43, PTEN and Csk in astrocytes. The distribution of these proteins (Figure 3C; Cx43 in red and PTEN or Csk in green) was analyzed using a confocal microscope. The yellow color in the overlay images confirmed certain areas of colocalization between Cx43 and PTEN or Csk in astrocytes (Figure 3C). This colocalization can be visualized in the Z-projection of the stack images (Figure 3C). In addition, C6 glioma cells were transiently transfected with Cx43, and PTEN (green) and Cx43 (red) were analyzed by immunofluorescence 48h later. The confocal images showed some points of colocalization (yellow) between the two proteins in the overlay image (Figure 3D).

### Region of Cx43 involved in the PTEN and Csk binding

**Figure 4:**
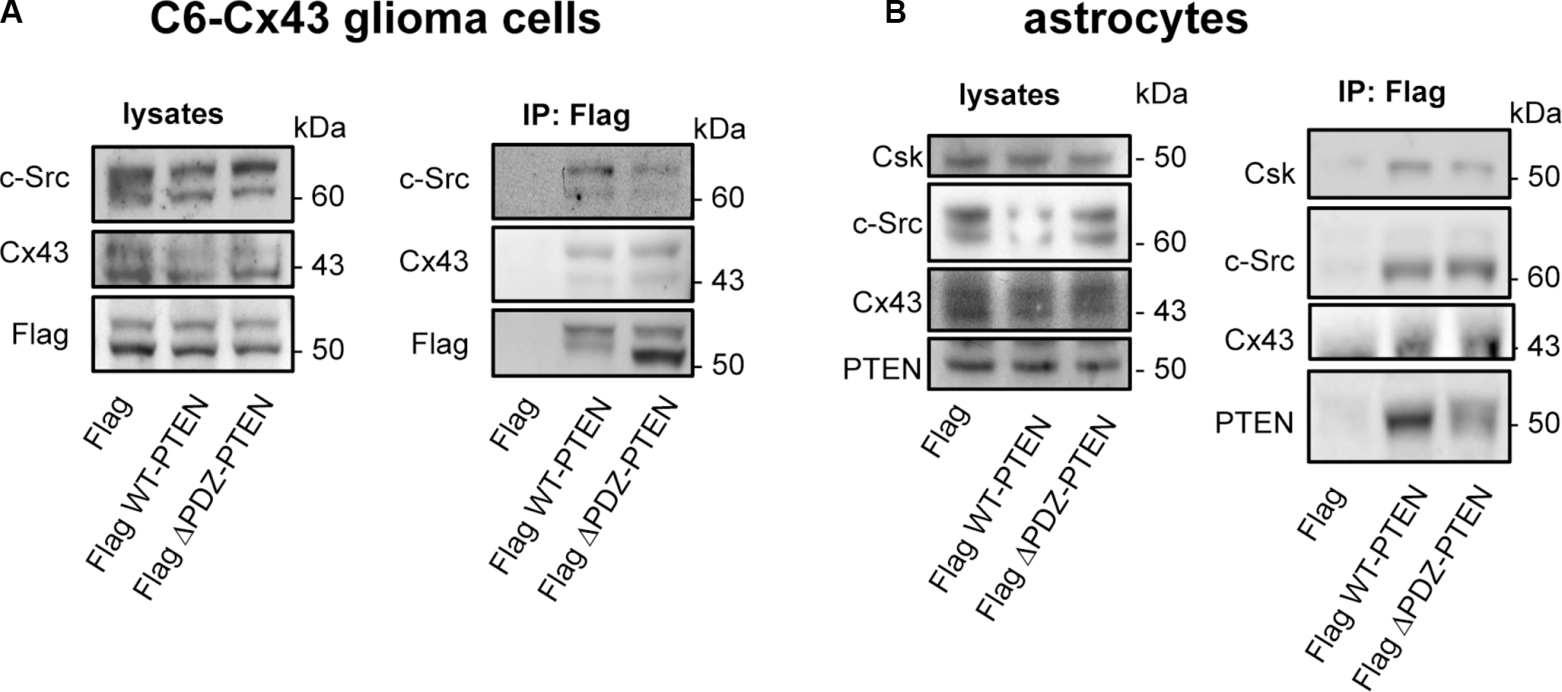
PTEN PDZ domain-binding motif is not required for PTEN interaction with Cx43, c-Src, and Csk C6-Cx43 cells (**A**) or astrocytes (**B**) were transfected with Flag WT-PTEN, Flag ΔPDZ-PTEN (1-400; PTEN lacking the PDZ domain-binding motif) or Flag (control). After 48 h, the cells were lysed, and immunoprecipitations were carried out with antibodies against Flag. Western blotting before (lysates) and after Flag immunoprecipitation for Flag, Cx43, c-Src, PTEN and Csk showing the presence of c-Src and Cx43 in the immunocomplex obtained with both WT-PTEN and ΔPDZ-PTEN in glioma cells expressing Cx43 and the presence of c-Src, Csk and Cx43 in the immunocomplex obtained with both WT-PTEN and ΔPDZ-PTEN in astrocytes that endogenously express Cx43.

Regarding the molecular bases underlying the Cx43-PTEN binding, both Cx43 and PTEN harbor a functional PDZ domain-binding motif (residues 380–382 in Cx43 [35] and residues 401–403 in PTEN [36]) at their C-terminal tail. Therefore, we hypothesized that Cx43 and PTEN could interact through a scaffolding protein containing several PDZ domains. For instance, Zonula occludens-1 (ZO-1) binds to Cx43 through one of its three PDZ domains [35]; therefore, although a direct interaction between ZO-1 and PTEN has not been reported, it might occur in one of the remaining PDZ domains in ZO-1. To test this concept, C6-Cx43 glioma cells or astrocytes were transfected with a mutant PTEN that lacks the PDZ domain-binding motif (residues 1–400; Flag ΔPDZ-PTEN), wild-type PTEN (Flag WT-PTEN) or the empty vector (Flag) (Figure 4). Flag immunoprecipitation revealed that the mutant PTEN retains the ability to bind to Cx43 and c-Src in glioma cells (Figure 4A) and to Cx43, c-Src and Csk in astrocytes (Figure 4B), indicating that the PDZ domain-binding motif is not required for these interactions.

**Figure 5:**
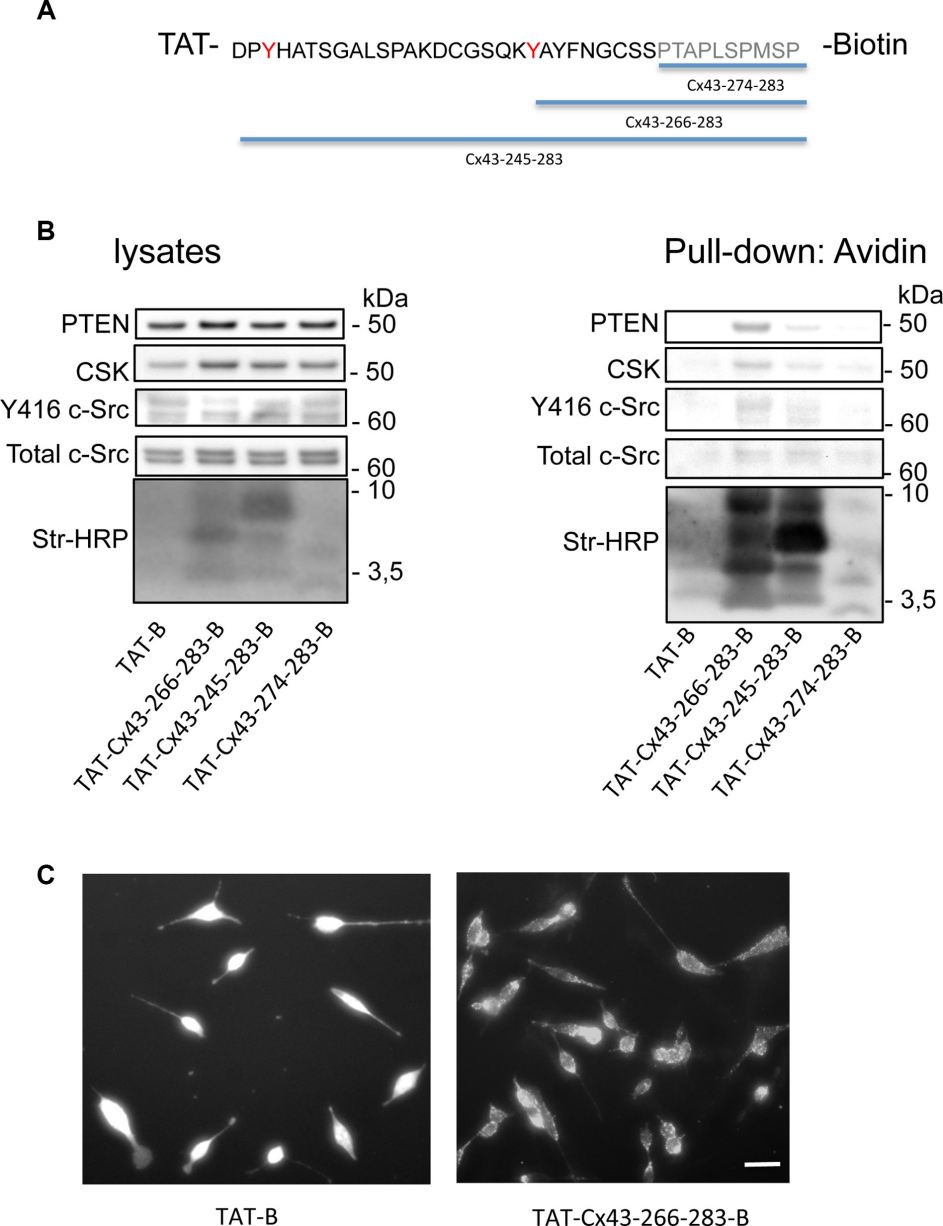
The Cx43 region involved in the recruitment of PTEN and Csk C6 glioma cells were incubated with several cell-penetrating peptides containing the indicated sequences of Cx43 fused to biotin for 30 min. (**A**) The SH3 domain binding motif is shown in grey and the tyrosines phosphorylated by c-Src in red. (**B**) After 30 min, the cells were lysed, and pull-down assays were carried out with avidin-conjugated agarose beads. Western blots before (lysates) and after avidin pull-down for c-Src, Csk and PTEN showing the enrichment of PTEN and Csk in the complex obtained with TAT-Cx43-266-283-Biotin and, to a lesser extent, with TAT-Cx43-245-283-Biotin. Str-HRP, HRP-conjugated streptavidin. (**C**) After 30 min, the cells were fixed, and the uptake of peptides bound to biotin was analyzed by fluorescence microscopy. Scale bars: 15 μm.

Our previous results suggested that cell-penetrating peptides (CPPs) containing Cx43 residues involved in the c-Src interaction (residues 245–283 and 266–283 but not 274–283 in the C-terminal domain of Cx43 (Cx43CT)) inhibit the activity of c-Src [11]. Therefore, we investigated whether these peptides could recruit the machinery required to inhibit c-Src activity. To address this point, C6 glioma cells were incubated with biotinylated CPPs containing the SH3 domain binding motif and tyrosines phosphorylated by c-Src (from amino acid 245 to 283), the same sequence excluding the tyrosines (from amino acid 266 to 283) or a consensus SH3 domain binding motif (from amino acid 274 to 283) (Figure 5A). These peptides were all fused to the TAT penetrating sequence (YGRKKRRQRRR). After 30 minutes, pull-down assays were performed, the biotinylated peptides were recovered and proteins bound to them were analyzed (Figure 5B). Our results showed that as expected [37], c-Src was found in complex with TAT-Cx43-266-283-B, TAT-Cx43-245-283-B and, to a lesser extent, TAT-Cx43-274-283-B. Interestingly, PTEN and Csk were mainly bound to TAT-Cx43-266-283-B and, to a lesser extent, TAT-Cx43-245-283-B, suggesting that this region of Cx43 (266–283) recruits not only c-Src but also PTEN and Csk. These proteins were not found when the cells were incubated with TAT-B, confirming that the sequence of Cx43 containing the residues 266–283 is sufficient to recruit c-Src, PTEN and Csk. The cellular internalization of these peptides was confirmed by fluorescence microscopy (Figure 5C).

### TAT-Cx43-266-283 inhibits human glioblastoma stem cell growth in a PTEN-dependent fashion

To confirm that the recruitment of PTEN and Csk to the sequence of Cx43 containing residues 266–283 is sufficient to inhibit c-Src and its downstream pathway, we tested the effect of the peptide TAT-Cx43-266-283 on G166 human glioblastoma stem cells (GSCs) [38].

**Figure 6:**
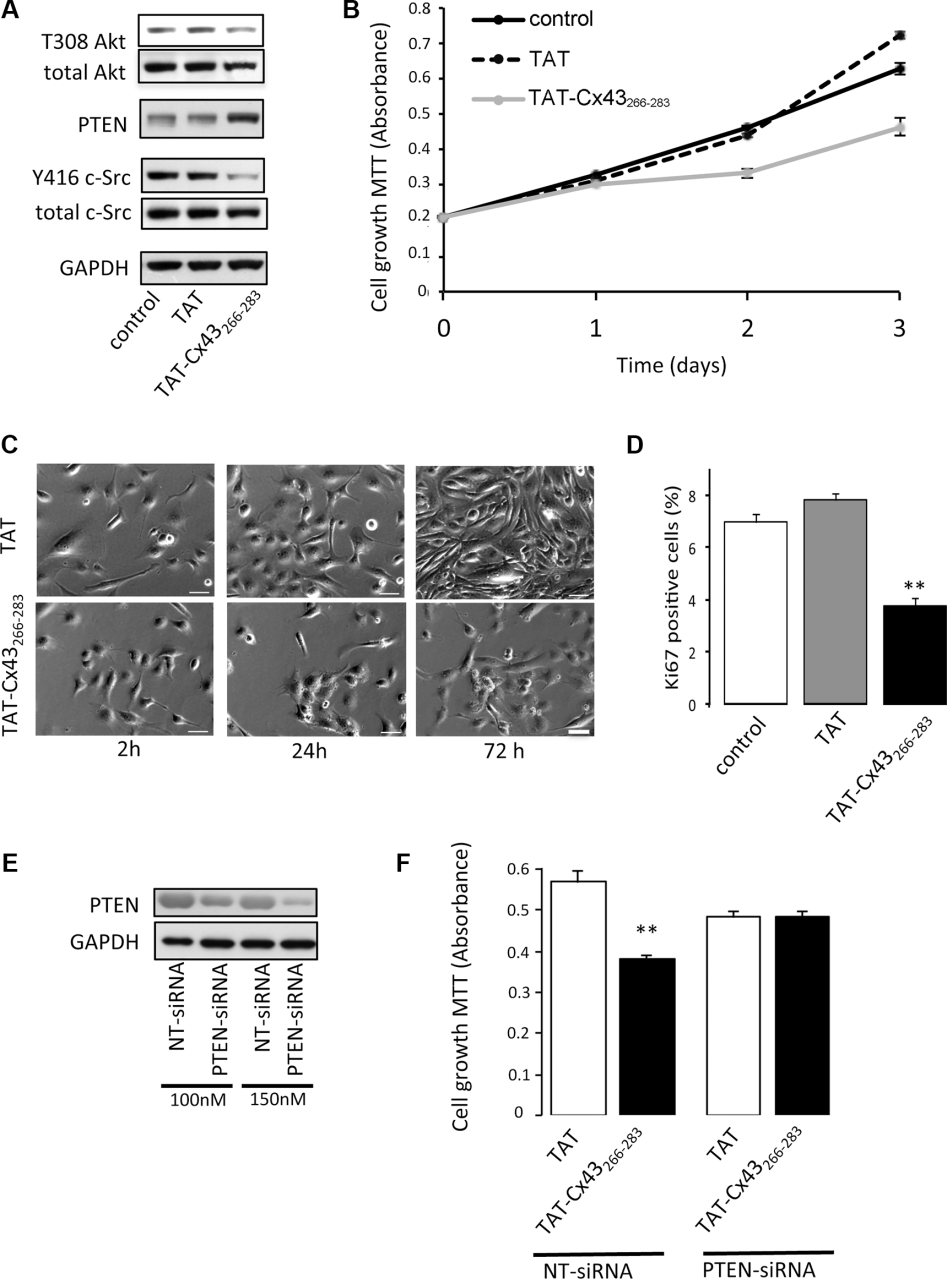
Involvement of PTEN in the antiproliferative effect of TAT-Cx43-266-283 on G166 human glioblastoma stem cells G166 cells were incubated with 50 μM TAT or TAT-Cx43-266-283 for the indicated times. (**A**) After 48 h, total c-Src, Y416 c-Src, PTEN, total Akt and T308 Akt levels were analyzed by Western blotting. (**B**) The cells were plated at a density of 5000 cells/cm^2^ and the number of living cells was monitored over a 3 day period by the MTT assay. Means ± s.e.m. (*n* = 3). (**C**) Representative phase-contrast images showing the inhibition of proliferation promoted by TAT-Cx43-266-283. Scale bars: 50 μm. (**D**) Percentage of Ki-67-positive cells found after 48h. Means ± s.e.m (*n* = 3). ** *p* < 0.01 versus control. (**E**) G166 cells were plated at 7500 cells/cm^2^ and transfected with PTEN-siRNA or non-targeting siRNA (NT-siRNA). After 48 h, PTEN levels were analyzed by Western blotting. (**F**) G166 cells were plated at 7500 cells/cm^2^ and transfected with 150 nM NT-siRNA or PTEN-siRNA. After 24 h, the cells were incubated with 50 μM TAT or TAT-Cx43-266-283 for 48 h and the number of living cells was analyzed by the MTT assay. Means ± s.e.m. (*n* = 3). **p < 0.01 versus TAT NT-siRNA.

Our results showed that TAT-Cx43-266-283 reduced c-Src activity as evidenced by decreased levels of Y416 c-Src, increased PTEN protein levels and subsequently reduced Akt activity (T308 Akt) (Figure 6A).

As a consequence of the inhibition of these proliferative pathways, TAT-Cx43-266-283 reduced GSC proliferation compared with the control or TAT peptides (Figure 6B–6D and Supplementary Figure S1). Interestingly, when PTEN was knocked-down by siRNA (Figure 6E), TAT-Cx43-266-283 was not able to significantly affect GSC growth. This result was in contrast to the observations made in GSCs transfected with a NT-siRNA (Figure 6F) and confirms the participation of PTEN in the inhibition of c-Src that is promoted by the sequence of Cx43 containing the residues 266–283.

## DISCUSSION

High-grade glioma cells exhibit reduced levels of Cx43 protein [3–9] but increased c-Src activity [20]. Importantly, restoring Cx43 expression inhibits the activity of c-Src [11, 19], reduces glioma cell proliferation [39, 40] and reverses glioma stem cell phenotype [11, 12]. In this study, we revealed the mechanism by which Cx43 inhibits the oncogenic activity of c-Src. Our results showed that Cx43, in addition to c-Src, recruits Csk and PTEN, which are the enzymes required to inhibit c-Src activity.

The Csk-mediated phosphorylation of c-Src at tyrosine 527 is the best-described mechanism for c-Src inactivation [41]. The completion of this inhibition requires the dephosphorylation of c-Src at tyrosine 416 [42]. Our results suggest that in C6 glioma cells, as in other cell types [28, 43], PTEN is the phosphatase that catalyzes this process. Indeed, silencing PTEN prevented the reduction of Y416 c-Src that was promoted by Cx43 in glioma cells. In addition, when either Csk or PTEN was silenced and c-Src activity could not be inhibited, the antiproliferative effect of Cx43 on glioma cells was reduced. It can be concluded that Csk and PTEN participate in the antiproliferative effect of Cx43 by inhibiting c-Src.

**Figure 7:**
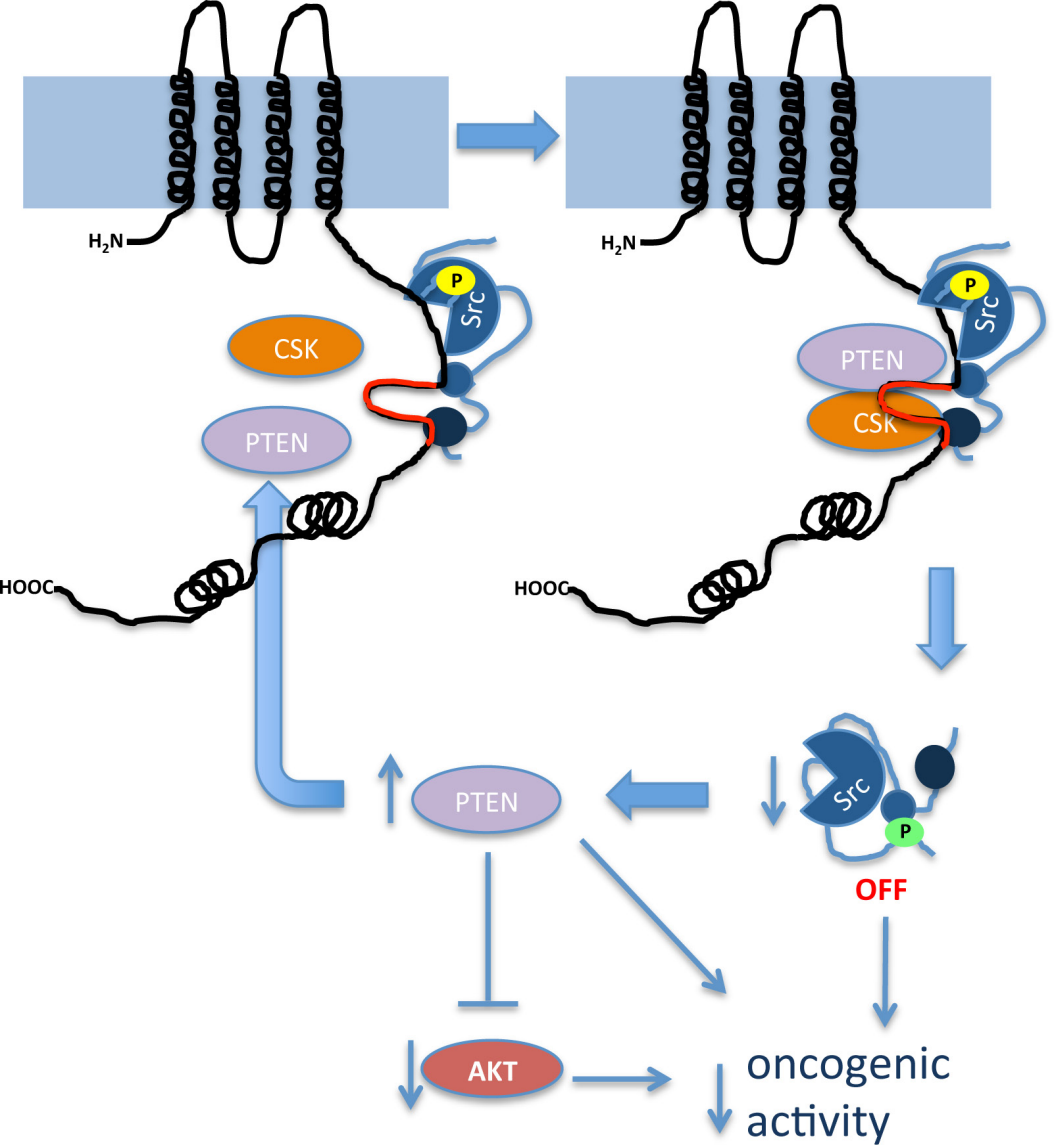
Proposed mechanism by which Cx43 inhibits the activity of c-Src Our results showed that the region of Cx43 that comprises amino acids 266-283 (in red), located in one of the intrinsically disordered regions of Cx43CT, serves as a docking platform for the active form of c-Src (c-Src phosphorylated at tyrosine 416, shown in yellow), Csk and PTEN. The proximity of these proteins facilitates the phosphorylation of c-Src at tyrosine 527 (green) catalyzed by Csk and the removal of phosphate at tyrosine 416 (yellow) catalyzed by PTEN with the subsequent inhibition of c-Src activity. c-Src inhibition prevents PTEN degradation creating a positive feed-forward loop to guarantee the long-term regulation of this process. As a consequence of increased PTEN, Akt activity decreases. This signaling pathway contributes to a reduction in the oncogenic activity of these proteins in glioma cells.

Intriguingly, our results showed that restoring Cx43 expression in glioma cells up-regulates the levels of functionally active PTEN protein. Indeed, PI3K/Akt, the main PTEN downstream pathway, is subsequently inhibited. Because the mutant Cx43 that lacks the ability to inhibit c-Src activity [19] did not modify PTEN levels or activity, it could be proposed that the effect of Cx43 on PTEN levels is a consequence of c-Src inhibition. In agreement with this proposal, the activity of c-Src promotes PTEN degradation and the subsequent activation of the PI3K/Akt pathway [33]. By showing that Cx43 up-regulates PTEN, one of the most relevant tumor suppressor genes in gliomas [44, 45], and inhibits PI3K/Akt, an important proliferative pathway that is frequently up-regulated in glioma [46], these results expand our knowledge of the mechanism by which Cx43 suppresses tumor growth (Figure 7).

This study shows that in astrocytes, that express high levels of endogenous Cx43, there is also an interaction between Cx43, c-Src, Csk and PTEN, indicating that the regulation of c-Src activity is a physiological function of Cx43. c-Src phosphorylates tyrosines 265 and 247 [14], which strongly affects Cx43 structure and function [26, 37] and the interaction of Cx43 with several partners [47–49]. To terminate these effects, the phosphatase TC- PTP is recruited to remove the phosphates at tyrosines 265 and 247 of Cx43 [50]. However, if c-Src remained active, it would continue to phosphorylate Cx43. Our results suggest that Cx43 phosphorylation by c-Src could also be a signal to recruit PTEN and Csk to this complex. The inhibition of c-Src activity promoted by PTEN and Csk would collaborate with TC-PTP to terminate this pathway, which is required to recycle Cx43 [51]. In this way, the transient and dynamic interactions of these proteins would contribute to the maintenance of the homeostasis of the gap junction channel and hemichannel activities.

Although both PTEN and Cx43 contain PDZ-binding motifs, our results indicated that these regions are not required for the interaction between these proteins. Instead, the region of Cx43 that is involved in its interaction with c-Src (residues 266–283) is sufficient to recruit PTEN and Csk. In agreement with this finding, CPPs containing this sequence (TAT-Cx43-266-283) inhibited the oncogenic activity of c-Src [11], increased PTEN, reduced Akt activity and subsequently decreased human glioblastoma stem cell proliferation.

Since it was first described, the relationship between Cx43 and cancer has been deeply investigated [52]. However, it should be noted that although restoring Cx43 expression in glioma cells reduces proliferation, it can also have detrimental effects due to gap junctional communication or cytoskeletal interactions [53–56]. There is interest in the short sequence (residues 266–283) of Cx43 because it inhibits the oncogenic activity of c-Src and lacks the ability to interact with the cytoskeleton or to form gap junction channels. Nevertheless, unexpected effects cannot be ruled out. Therefore, further studies should be carried out to explore the therapeutic potential of this sequence in depth.

In conclusion, we propose that the Cx43-mediated inhibition of c-Src involves the recruitment of the c-Src inhibitors Csk and PTEN to residues 266–283 within Cx43CT. Furthermore, upon c-Src inhibition, Cx43 up-regulates PTEN and subsequently inactivates Akt to sustain its tumor suppressor properties (Figure 7). These results stress the relevance of Cx43 residues 266–283 for the development of new therapies to reduce the oncogenic activity of c-Src. In contrast to the available c-Src inhibitors, this approach uses an endogenous inhibitory mechanism, making off-target effects less likely.

## MATERIALS AND METHODS

### Cell cultures

Astrocytes in primary culture were prepared from the forebrains of 1- to 2-day-old Wistar rats and cultured in DMEM (Sigma-Aldrich Química, Madrid, Spain) supplemented with 10% FCS (Gibco, Life Technologies, Madrid, Spain), as previously described [57]. C6 glioma cells (ATCC, Manassas, USA) were cultured in DMEM supplemented with 10% FCS as previously described [19]. The G166 human GSC line was obtained from BioRep (Milan, Italy). The cells were grown in petri dishes coated with 10 mg/ml laminin (Life Technologies). The growth medium was RHB-A (StemCells, Cambridge, UK) supplemented with 1% N2, 2% B27 (Life Technologies), 20 ng/ml EGF and 20 ng/ml b-FGF (PeproTech, London, UK) as described previously [11].

### Plasmid constructs and cell transfection

The pIRES-Cx43 construct was generated as previously reported [19] by ligating a PCR-amplified fragment encoding the rat Cx43 sequence (NM_012567) into the *Xho*I-*Bam*HI sites of the bicistronic pIRES2-DsRed2 vector (Clontech, Palo Alto, CA, USA). The Cx43Y247F/Y265F mutation was introduced into pIRES-Cx43 by site-directed mutagenesis [19]. C6-Ires, C6-Cx43 and C6-Cx43 Y247F/Y265F clones were generated and characterized as previously described [19]. Unless otherwise specified, the C6 glioma cells were stably transfected with the empty vector (C6-Ires), the construct containing Cx43 (C6-Cx43) or the construct containing the mutant Cx43 (C6-Cx43Y247F/Y265F) using Lipofectamine 2000 (Life Technologies). For stable transfection cells were selected with 0.5 mg/ml G418 (Promega, Madison, WI, USA) in DMEM supplemented with 10% (v/v) FCS.

The pSG5L, pSG5L HA-PTEN, pCMV FLAG WT-PTEN and pCMV FLAG ΔPDZ-PTEN (1–400) plasmids were a gift from William Sellers and Hong Wu and were obtained from Addgene (Addgene plasmid 10737, 10750, 22231 and 22232, respectively) [58, 59]. Cells were transiently transfected using Lipofectamine 2000, as described above.

### Transfection of siRNA

C6-Cx43 clone 7 cells were transfected with a validated non-targeting siRNA (NT-siRNA), a siRNA specific for Csk (Csk-siRNA) or a siRNA specific for PTEN (PTEN-siRNA) (BioNova Científica S.L., Madrid, Spain). Cells were transfected with the double-strand siRNA complexed with 3 μl/ml Lipofectamine 2000 (Life Technologies) in culture medium without antibiotics. The cells were maintained in the presence of the oligonucleotides in culture medium without antibiotics for 6 h. The extent of siRNA-mediated down-regulation of Csk or PTEN expression was evaluated by Western blot analysis. The Csk-siRNA sequences were as follows: sense 5′-AGUACCCAGCAAAUGGGCATT-3′ and antisense 5′-UGCCCAUUUGCUGGGUACUTT-3′ [29]. The PTEN-siRNA sequences were as follows: sense 5′-GUUAGCAGAAACAAAAGGAGATT-3′ and antisense 5′-UCUCCUUUUGUUUCUGCUAACTT-3′ [30].

### Peptide treatments

The synthetic peptides (> 90% pure) were obtained from GenScript (Piscataway, NJ, USA). All peptides used were biotinylated via a C-terminal lysine. YGRKKRRQRRR-Lys(biotin) was used as the TAT sequence (TAT-B), which is responsible for cell penetration of the peptides [60]. The sequence for TAT-Cx43-245-283-B was YGRKKRRQRRRDPYHATSGALSPAKDCGSQKYAYFNGCSSPTAPLSPMSP-Lys(biotin), the sequence for TAT-Cx43-266-283-B was YGRKKRRQ RRRAYFNGCSSPTAPLSPMSP-Lys(biotin) and the sequence for TAT-274-283-B was YGRKKRRQRRR PTAPLSPMSP-Lys(biotin). Peptides were used at 50 μM in culture medium at 37 °C for the indicated times.

### MTT assay

Cells cultured at 37°C in 24-well-plates were incubated in the dark for 75 min with 300 μl of DMEM containing 0.5 mg/ml MTT. The medium was then removed, and the cells were incubated for 10 min in the dark with dimethyl sulfoxide (500 μl/well). Finally, the absorbance was measured at a wavelength of 570 nm using a microplate reader (Appliskan 2001; Thermo Electron Corporation, Waltham, MA, USA).

### Western blot analysis

Western blotting was performed as previously described [11]. Briefly, equivalent amounts of proteins (20 *μ*g per lane) were separated on NuPAGE Novex Bis-Tris (4–12% or 10%) midigels (Life Technologies). Proteins were transblotted using an iBlot dry blotting system (Life Technologies). The membranes were cut into several strips to be immunoblotted with distinct antibodies, thus allowing for comparative analysis of the amount of each protein in the same sample. Membranes were then blocked for 1 h at room temperature in Tris-buffered saline containing 0.05% Tween 20 (TTBS) and 7% non-fat milk powder before being incubated overnight at 4 °C with the primary antibodies. The primary antibodies used were: Cx43 (1:250; BD Biosciences, Madrid, Spain; Ref. 610062); total c-Src (1:500; Ref. 2108); Y416 c-Src (1:250; Ref. 2101), Y527 c-Src (1:500; Ref. 2105), Csk (1:500; Ref. 4980), PTEN (1:500; Ref. 9552), total Akt (1:1000; Ref. 9272), S473 Akt (1:2000; Ref. 4060), T308 Akt (1:1000; Ref. 4056) (all from Cell Signaling, Danvers, MA, USA), GAPDH (1:6000; Ambion; Ref. AM4300) or *α* -actinin (1:1000; Merck Millipore, Darmstadt, Germany; Ref. MAB1682). After extensive washing, the membranes were incubated with HRP-conjugated antibodies (Santa Cruz Biotechnology, Inc., Dallas, TX, USA; Refs. sc-2030 and sc-2005) in TTBS. The proteins were developed with a chemiluminescent substrate. Densitometry analysis of the bands was performed using the Image J program (Wayne Rasband; NIH, Bethesda, MD, USA). The amounts of GAPDH or *α*-actinin recovered in each sample served as the loading control, and the values for each protein were normalized to their corresponding GAPDH or *α*-actinin level.

### Immunofluorescence

Immunofluorescence was performed as previously described [11]. The cells were incubated overnight at 4°C with primary antibodies against Cx43 (1:100; BD Biosciences; Ref. 610062), Csk (1:100), and PTEN (1:100) (both from Cell Signaling; Refs. 4980 and 9552). The secondary antibodies used were as follows: Cy5 goat anti-mouse (1:500; Jackson ImmunoResearch, Baltimore, PA, USA; Ref. 115-175-003) and Alexa Fluor 488 goat anti-rabbit (1:1000; Life Technologies, Ref. A-11029). The cells were mounted using the SlowFade Gold Antifade kit (Life Technologies) and were analyzed using a Leica DM-IRE2 confocal microscope and LCS Lite software (Leica Microsystems, Wetzlar, Germany).

### Co-immunoprecipitation

Twenty-one DIV astrocytes or C6 cells grown to confluence in 10-cm dishes were washed with ice-cold PBS and lysed at 4°C in 1 ml of SDS-free RIPA buffer (10 mM Tris-HCl (pH 8.0), 150 mM NaCl, 0.2% Triton X-100, 2 mM EDTA, 2 mM EGTA, 1 mM PMSF, 1:100 (v/v) protease cocktail (Cocktail III; Calbiochem Merck-Millipore, Billerica, MA, EE.UU), 1 mM NaF and 0.1 mM Na_3_VO_4_). Lysates were centrifuged at 11000 × *g* for 10 min at 4°C, and the supernatants were recovered. A 25-μl aliquot was used to analyze the protein content (lysates), and the remaining lysate (immunoprecipitation) was incubated with 2 μg of mouse monoclonal antibody against hexokinase I (Merck Millipore; Ref. MAB1532), mouse monoclonal antibody against HA (Roche, Basel, Switzerland Ref.11 583 816 001), mouse monoclonal antibody against Cx43 (BD Biosciences; Ref. 610062), rabbit monoclonal antibody against Csk (Cell Signaling; Ref. 4980) or rabbit monoclonal antibody against GAPDH (Ambion; Ref. AM4300) for 12 h at 4°C with gentle shaking. The immunocomplexes were sequestered by adding 50 μl of Protein-A Sepharose CL-4B (GE Healthcare, Madrid, Spain), which was previously saturated with 5% (w/v) albumin and gently shaken at 4°C for 4 h. The Protein-A beads containing the immunocomplexes were collected by centrifugation (11000 × *g* for 1 min at 4°C). The beads were then washed four times with buffer A (10 mM Tris-HCl (pH 8.0), 150 mM NaCl, Triton X-100 and 2 mM EDTA), once with buffer B (10 mM Tris-HCl (pH 8.0), 500 mM NaCl, 0.2% Triton X-100 and 2 mM EDTA) and once with buffer C (10 mM Tris-HCl (pH 8.0)). The bound proteins were eluted with SDS sample buffer at 95°C for 5 min. After centrifugation, the supernatants were analyzed by Western blotting.

For Flag fusion proteins, the lysis buffer contained 20 mM Tris-HCl (pH 8.0), 137 mM NaCl, 1% IGEPAL (p/v), 1 mM PMSF, 1:100 (v/v) protease cocktail (Cocktail III, Calbiochem), 1 mM NaF and 0.1 mM Na_3_VO_4_. The lysates were incubated with anti-Flag M2 Affinity Gel (40 μl per ml; Sigma, Saint Louis, MO, USA; Ref.F7425) for 12 h at 4°C with gentle shaking. The anti-Flag beads containing the immunocomplexes were collected by centrifugation (11000 × *g* for 1 min at 4°C) and washed five times with lysis buffer, and the bound proteins were eluted and analyzed by Western blotting.

### Avidin pull-down assay

Cells grown in 3.5-cm dishes were incubated with 50 μM biotinylated peptides for 30 min. Proteins were then collected in 1 ml of lysis buffer (20 mM Tris-HCl (pH 8.0), 137 mM NaCl, 1% IGEPAL, 1 mM PMSF, protease cocktail (1:100; Cocktail III, Calbiochem), 1 mM NaF and 0.1 mM Na_3_VO_4_). Lysates were centrifuged at 11000 × *g* for 10 min at 4°C, and the supernatants were recovered. A 25-μl aliquot of each lysate was used to analyze the protein content, and the remaining lysate was incubated with NeutrAvidin-Agarose (Thermo Scientific, Rockford, IL, USA; Ref. 29200) for 12 h at 4°C with gentle shaking. The avidin beads bound with the peptides were collected by centrifugation (3000 × *g* for 1 min at 4°C). The beads were then washed five times with lysis buffer, and the bound proteins were eluted and analyzed by Western blotting. To detect biotinylated peptides, the membranes were incubated with HRP-conjugated streptavidin in TTBS (1:40000, Ref. 434323, Life Technologies) and then developed with a chemiluminescent substrate.

In parallel, the cells were incubated with 50 μM biotinylated peptides for 30 min. The cells were then washed with PBS at 4°C and fixed with 4% paraformaldehyde for 20 min. After washing, the cells were incubated with Cy2-conjugated streptavidin (1:500; Jackson ImmunoResearch, Baltimore, USA; Ref. 016-220-084) for 1 h, mounted and visualized as described previously.

### Statistical analyses

The results were expressed as the means ± s.e.m. of at least three independent experiments. Statistical analyses were carried out using Student's *t*-test when two groups were compared. For the comparison of more than one group, analysis of variance (one-way ANOVA) was used, followed by an appropriate post -test (Dunnet or Tukey). Values were considered significant with a *p* value less than 0.05.

## SUPPLEMENTARY MATERIALS FIGURES


