# Supplementary material for: Connexin43 recruits PTEN and Csk to inhibit c-Src activity in glioma cells and astrocytes
Source: Oncotarget. 2016 Jul 6;7(31):49819–33. doi: 10.18632/oncotarget.10454 (PMC5226550; doi:10.18632/oncotarget.10454)
Supplement: Supplementary file 1 [file oncotarget-07-49819-s001.pdf]

Connexin43 recruits PTEN and Csk to inhibit c-Src activity in glioma cells and astrocytes

Supplementary Materials

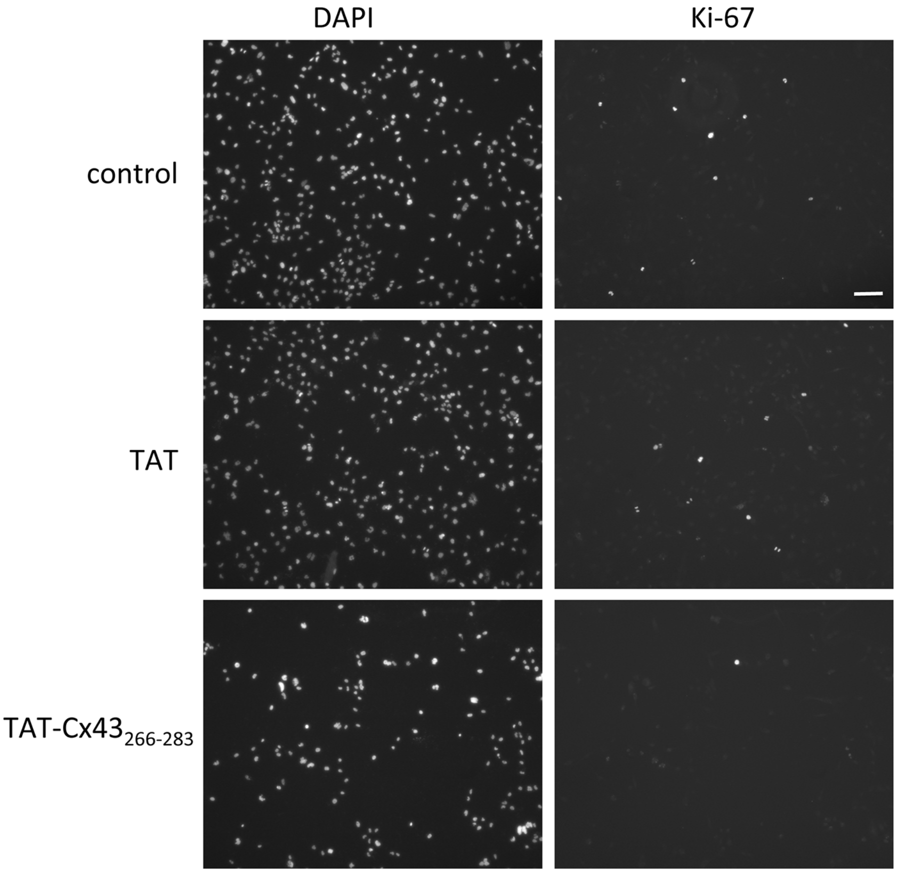

Supplementary Figure S1: Effect of TAT-Cx43-266-283 on the expression of Ki-67 in G166 human glioblastoma stem cells (GSCs). G166 cells were incubated with TAT or TAT-Cx43-266-283 for 48 h. The DAPI and Ki-67 photomicrographs are of the same field and show the reduction of Ki-67-positive cells promoted by TAT-Cx43-266-283. Scale bar = 50 µm.
